# Multiple Acyl-CoA Dehydrogenase Deficiency: A Rare Cause of Hepatomegaly

**DOI:** 10.14309/crj.0000000000001036

**Published:** 2023-05-06

**Authors:** Raissa Nana Sede Mbakop, Arnold Nongmoh Forlemu, Wuttiporn Manatsathit

**Affiliations:** 1 Division of Gastroenterology, Department of Internal Medicine, Piedmont Athens Regional, Athens, GA; 2 Division of Gastroenterology and Hepatology, Department of Internal Medicine, The Brooklyn Hospital Center, Brooklyn, NY; 3 Division of Gastroenterology and Hepatology, Department of Internal Medicine, Omaha, NE

**Keywords:** MADD, liver disease, hypoglycemia, riboflavin, acylcarnitine

## Abstract

Multiple Acyl-CoA dehydrogenase deficiency (MADD) is an autosomal recessive disorder that can manifest with hepatic and muscular dysfunction. MADD can be fatal in neonates; however, late-onset MADD has a milder course and often becomes symptomatic during adulthood. A 20-year-old patient presented to the hepatology clinic with elevated liver enzymes and hepatomegaly. Several investigations including a liver biopsy were unremarkable. Subsequently, the patient developed rhabdomyolysis and nonketotic hypoglycemia raising suspicion for mitochondrial disorders. Plasma acylcarnitine levels performed showed elevated C4-C18:2 consistent with MADD. Although the patient denied a complete genetic evaluation, the patient had complete resolution of symptoms after riboflavin and diet modification.

## INTRODUCTION

Multiple Acyl-CoA dehydrogenase deficiency (MADD) is a severe inborn error of lipid metabolism that can present with hypoketotic hypoglycemia, myopathy, chronic nausea and vomiting, and liver dysfunction,^1^ occasionally progressing to liver failure or cirrhosis.^2,3^ Electron transfer flavoprotein dehydrogenase (ETFDH) is a key protein located in the inner membrane of mitochondria and mediates electron transport from flavoprotein dehydrogenases to the ubiquinone pool as part of the electron transport chain for energy production.^4^ ETFDH deficiency disrupts mitochondrial electron transfer, lipid storage, and amino acid and choline metabolism. We discuss a patient who was found to have riboflavin-responsive late-onset MADD or type III MADD.

## CASE REPORT

**Figure 1.**
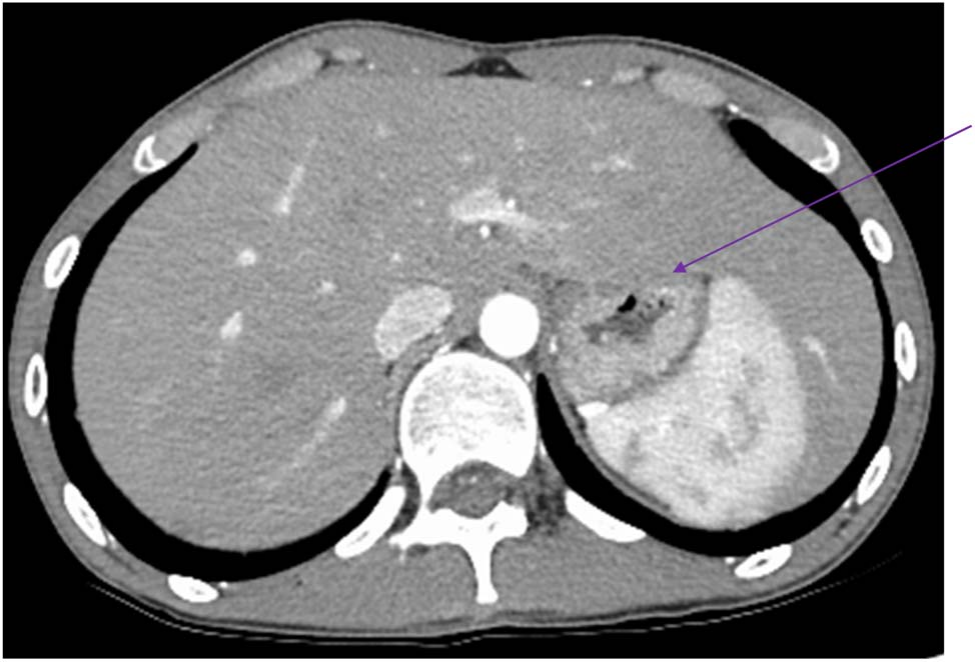
Severe hepatomegaly with an enlarged left liver lobe compressing the stomach.

A 20-year-old Asian man with no history of alcohol use presented to the hepatology clinic for elevated liver enzymes. He was admitted 2 months prior for diffuse muscle aches, 20-lb unintentional weight loss, elevated aspartate aminotransferase at 380 U/L, and alanine aminotransferase at 117 U/L. An abdominal contrast computed tomography revealed a markedly enlarged liver compressing the stomach (Figure 1). The workup for viral hepatitis (hepatitis A immunoglobulin [Ig] G and IgM, hepatitis B surface antigen, antibody and core, hepatitis C and E antibodies), autoimmune hepatitis (anti-nuclear antibody, anti-smooth muscle antibody, anti-liver kidney microsomal antibody), Wilson disease (ceruloplasmin, 24-hour urinary copper), celiac disease (tissue transglutaminase IgA, IgA total), alpha-1-antitrypsin deficiency level, hemochromatosis (iron study with ferritin level), thyroid disease (thyroid-stimulating hormone), malignancy, and other infectious etiologies was all negative. A liver biopsy showed rare glycogenated nuclei, but electron microscopy was negative for glycogen storage disease. During the hospitalization, the liver function test normalized after intravenous fluid.

One month after the clinic visit, he presented with proximal muscle weakness and was found to have severe rhabdomyolysis with creatinine kinase of 12,449 U/L and low glucose at 50 mg/dL. Electrophysiology studies showed diffuse myopathy without evidence of neuropathy. The patient's nonketotic hypoglycemia and myopathy raised a concern for mitochondrial disorder; hence, we obtained free and total carnitine levels that were low (9 and 24 μmol/L, respectively). Urine organic acids showed elevated ethylmalonic, pyruvic, and suberic acids. An acylcarnitine profile was then obtained, which showed moderate elevations of short-chain, medium-chain, and long-chain acylcarnitines suggestive of late-onset MADD (C4 to C18:2 with the highest value for C10) (Table 1). Genetic testing showed 2 heterozygous mutations in ETFDH genes [c.560 C>T, p.(A187V) and c.1669 G>A, p.(E557K)]. Additional genetic testing was recommended; unfortunately, the patient did not want to pursue additional testing. Riboflavin was initiated (50 mg 3 times daily for 1 week followed by 100 mg 3 times daily) with complete resolution of the symptoms. Therefore, he was diagnosed with late-onset or type III MADD based on the acylcarnitine profile and complete resolution after a trial of riboflavin. He eventually gained about 17 lbs and did not develop recurrence of rhabdomyolysis or hypoglycemia.

**Table 1.** Serum carnitine, acyl carnitine, and urine organic acid profiles

Parameter	Value	Reference range
Free carnitine, μmol/L	9	22–63
Total carnitine, μmol/L	24	31–78
Acylcarnitine profile, μmol/L		
C2, Acetyl	6.4	2.9–15.1
C3, Propionyl	0.4	≤0.8
C4, Iso-/Butyryl	0.5	≤0.4
C5, Isovaleryl/2-Mebutyryl	0.3	≤0.2
C5-DC, Glutaryl	0.2	≤0.2
C5-OH, 3-OH Isovaleryl	<0.01	≤0.07
C6, Hexanoyl	0.6	≤0.1
C8, Octanoyl	1.4	≤0.2
C8:1, Octenoyl	0.3	≤0.6
C10, Decanoyl	1.9	≤0.3
C10:1, Decenoyl	0.4	≤0.3
C12, Dodecanoyl	0.9	≤0.1
C12:1 Dodecenoyl	0.2	≤0.1
C12-OH, 3-OH-Dodecanoyl	<0.01	≤0.01
C14, Tetradecanoyl	0.6	≤0.1
C14:1, Tetradecenoyl	0.8	≤0.2
C14:2 Tetradecadienoyl	0.3	≤0.1
C14-OH, 3-OH-Tetradecanoyl	<0.01	≤0.02
C14:1-OH, 3-OH-Tetradecenoyl	0.01	≤0.04
C16, Palmitoyl	0.6	≤0.1
C16:1, Palmitoleyl	0.5	≤0.04
C16-OH, 3-OH-Palmitoyl	<0.01	≤0.02
C16:1-OH, 3-OH-Palmitoleyl	0.04	≤0.02
C18, Stearoyl	0.3	≤0.1
C18:1, Oleyl	0.4	≤0.2
C18:2, Linoleyl	0.3	≤0.1
C18-OH, 3-OH-Stearoyl	<0.01	≤0.02
Urine organic acids		
Pyruvic acid	20	0–15
Ethylmalonic acid	7	0–4
Suberic acid	4	0–3
All other acids tested	Not detected	

## DISCUSSION

MADD is caused by a deficiency of an electron transfer flavoprotein or an ETFDH. These defects lead to impaired adenosine triphosphate biosynthesis, excessive lipid accumulation in different organs, and insufficient gluconeogenesis.^1^ MADD's incidence at birth is estimated at 1 in 250,000.^5,6^ MADD can be classified into 3 subtypes. MADD type I and type II are often fatal homozygous null mutations that are neonatal in onset, appearing within 48 hours of life and manifest with or without congenital anomalies. MADD type III (late-onset MADD or riboflavin-responsive MADD), which was the case of this patient, is often mild, and the course and age of onset can be extremely variable. The clinical presentation of late-onset MADD can vary wildly with symptomatic episodes involving multiple organ symptoms, such as nausea/vomiting, hepatomegaly, myopathy, and encephalopathy.^7^ Some patients may present with chronic features such as muscle weakness and exercise intolerance, and muscle biopsy would reveal lipid storage myopathy. Patients with late-onset MADD often carry at least 1 missense variation with minor amounts of residual electron transfer flavoprotein/ETFDH activity, which is sufficient to prevent embryonic development of congenital anomalies.^1^ Unlike other types of MADDs, late-onset MADD diagnosis can be difficult because many patients may not display typical patterns of elevated urinary organic acids and serum acylcarnitine fatty acids during times of well-being.^5–7^ In plasma, acylcarnitines are characterized by elevations in short-chain, medium-chain, and long-chain acylcarnitines such as C4-C18:2. However, therapeutic interventions, such as triglyceride oil, valproic acid, or certain antibiotics, can mimic this biochemical profile.^7^ Therefore, the diagnosis of late-onset MADD should rely on blood and urine analysis and ultimate confirmation by molecular methods. Genetic tests in our case showed a pathogenic heterozygous mutation with 2 variants [c.560 C>T p.(A187V) and c.1669 G>A p.(E557K)] in ETFDH genes. c.560 C>T p.(A187V) has been shown to be pathogenic by a single submitter.^8^ The pathogenic significance of c.1669 G>A p.(E557K) is not known. Further genetic testing would likely have identified the nature of this variant; unfortunately, our patient did not pursue further genetic testing.

Late-onset MADD is typically very responsive to riboflavin (100–300 mg daily), carnitine, coenzyme Q_10_ supplements, and dietary changes.^1,4^ Patients admitted for acute decompensation of disease often require intravenous fluid containing at least 10% dextrose and bicarbonate therapy.

Myositis (autoimmune, inclusion body, sporadic) was raised as a differential diagnosis in our case, especially in the setting of proximal muscle weakness, elevated creatine kinase, and electrophysiology showing diffuse myopathy. However, exercise intolerance, hypoglycemia, and the acylcarnitine profile could not be explained by the varied forms of myositis. In addition, our patient's symptoms dramatically improved with riboflavin supplementation, supporting our diagnosis of late-onset MADD.

In conclusion, we present a patient with late-onset MADD which is often a milder form of MADD. The condition is rare and is predominant in individuals of Asian descent. The workup for late-onset MADD should be considered in patients with steatohepatitis unrelated to obesity and associated with elevated aspartate aminotransferase, alanine aminotransferase, nonketotic hypoglycemia, acidosis, and muscle involvement. The condition is treatable with timely initiation of dietary changes and riboflavin supplementation, which is lifesaving with improved quality of life.

## DISCLOSURES

Author contributions: All authors drafted, revised, reviewed literature, and equally contributed to approve the final manuscript. Arnold Nongmoh Forlemu is the article guarantor.

Financial disclosure: None to report.

Informed consent was obtained for this case report.
